# Multilocus Sequence Analysis of Selected Housekeeping- and Pathogenicity-Related Genes in *Venturia inaequalis*

**DOI:** 10.3390/pathogens10040447

**Published:** 2021-04-08

**Authors:** Monika Michalecka, Joanna Puławska

**Affiliations:** Department of Plant Protection, The National Institute of Horticultural Research, Konstytucji 3 Maja 1/3, 96-100 Skierniewice, Poland; joanna.pulawska@inhort.pl

**Keywords:** pathogenic race, phylogenetic inference, virulence

## Abstract

The relationship between housekeeping and pathogenicity-related genes and virulence or avirulence towards the primary *Malus* resistance genes (R) has not been previously studied for *Venturia inaequalis* fungus, the causal agent of apple scab. In this study, the sequences of two housekeeping genes encoding elongation factor alpha (EF-1α) and β-tubulin and two previously unstudied effector genes of *V. inaequalis* from mannosidase and glucosidase families of 100 strains collected from apple cultivars with *Rvi6*, *Rvi1*, and *Rvi17* and without known scab resistance genes were submitted to the analyses. Based on the phylogenetic and diversity data, as well as recombination analyses of the sequenced regions, we assessed the phylogenetic relationships and genetic structure of the pathogen within the species and the evolutionary forces that are currently acting upon this microorganism. The topology of the obtained phylograms demonstrates the lack of a relationship between the phylogenetic position of the strain and the host cultivar and the geographical origin or race of the strain. The isolates from different hosts were differentiated but did not form diagnosable, distinct phylogenetic groups. These results suggest that the analyzed genes may be too conserved to reflect the adaptation of pathogens to apple genotypes with different R genes; thus, they do not adequately reflect race discrimination. In contrast, based on variation and gene flow estimation, genetic divergence was observed among strains virulent to apple trees containing *Rvi6*. The results of this study confirmed a lack of free recombination between strains and demonstrated that the analyzed regions are in linkage disequilibrium and contain non-random polymorphisms associated with the strain.

## 1. Introduction

Apples are among the most economically important fruit tree crops in temperate zones, including Poland. The ascomycete *Venturia inaequalis* (Cooke) G. Winter is responsible for the most severe disease affecting apples, as well as many ornamental *Malus* species in many countries, including Poland: apple scab. Despite the consistent progress in breeding for resistance to apple scab, effective protection against the disease still primarily depends on repeated fungicide application in spring and leaf litter management in autumn [1]. To date, 17 pathogenic *V. inaequalis races* have been reported on *Malus* spp. [2], with each overcoming apple tree resistance related to one major gene of resistance (*R*). In recent decades, molecular population genetics have been widely utilized to demonstrate the patterns of genetic diversity within and between *V. inaequalis* populations and to trace the histories of divergence and speciation in pathogens by genotyping neutral microsatellite markers [3,4,5]. Several studies have obtained evidence that some races of the fungus possess unique genetic patterns recognized as an effect of strong selection pressure exerted by the host, as has been widely reported for the *Rvi6* apple gene [6,7,8,9,10,11]. It has been shown that *Rvi6* cultivars divide *V. inaequalis* populations into two genetically different subgroups, *Rvi6*-virulent (named race 6) and *Rvi6*-avirulent, while this structuring effect was not observed for other *R* genes. It is assumed that two pathogen lines exist: populations able to or not able to affect *Rvi6* hosts, respectively. These presumably diverged and were separated by distance long time ago [10]. Currently, secondary contact between these lineages with severely restricted gene flow is observed, thereby maintaining the genetic distance between them. If two separate lineages exist, nucleotide differences are also expected, at least in more variable fragments of genes connected with vital processes or in genes connected with the interaction with host or pathogenicity process. The influence of the apple genotype, including the main resistance genes (in this instance, *Rvi6*, *Rvi17* or *Rvi1*), on other genomic regions of *V. inaequalis*, including the core genome with housekeeping genes or genes encoding pathogenesis-related proteins, has not been studied to date.

The new gene combinations produced in the sexual stage are spread via abundantly produced spores of the asexual phase of fungus [1] which generates high genetic diversity within field populations, and is believed to be a factor facilitating pathogen adaptation to specific resistance genes of the host and environmental changes. Although pathogen variants (races) have been well characterized as causing difficulties in control, very little is known regarding genetic variation in regions of the *V. inaequalis* genome that are not under direct selection pressure from fungicides or from host resistance genes [12]. Aside from widely applied multilocus genotyping based on microsatellite markers, relatively few investigations have been undertaken in *V. inaequalis* to examine the changes in nucleotide diversity and to reveal intraspecies variations in this taxon. It was demonstrated that introns in the 18S rDNA gene and alleles of ITS1 provided genetic markers for subdividing populations of *V inaequalis* [12], while sequence analysis of the complete internal transcribed spacer region together with mating tests enabled the consideration of two formae speciales belonging to *V. inaequalis* that are responsible for scab on *Malus* and *Pyracantha* [13]. Analysis of the sequence data of six nuclear loci (actin, EF1α, the gene encoding the GTP-binding protein and the gene encoding a hypothetical protein conserved among fungi) of *V. inaequalis* strains derived from apple, pyracantha and loquat demonstrated that the strains were differentiated but did not form distinct phylogenetic species [7]. In contrast, no study has been performed to investigate whether the diversity of sequences of genes serving as phylogenetic markers or pathogenicity-related genes supports the separation between *Rvi6*-virulent and -avirulent *V. inaequalis* populations to date, as was reported from multilocus microsatellite loci analyses [11]. In parallel studies comparing gene expression profiles of *V. inaequalis* fungus growing in vitro and in planta [14], the upregulation of some genes coding cell wall-degrading enzymes (CWDEs) during fungal growth in planta was noted. Among these contigs, two regions encoding potentially pathogenicity-related genes were selected for sequence analysis.

Multilocus sequence analysis (MLSA) is a technique that enables the recognition of phylogenetic relationships between taxa based on genic regions. As reported by Hanage et al., 2006, the genes selected for the MLSA approach should be ubiquitous to the studied taxon and present in a single copy within the genome [15]. Several studies have been published describing the application of MLSA in the inferring of phylogeny based on pathogenicity factors [16], in the characterization of natural genetic variation and reproductive mode [17] or taxonomic delimitation [18] of plant and human pathogenic fungi. MLSA was also applied to discrimination between groups within the fungal species, by resolving pathogen population structure [16,18], and by determining genetic distinctness between closely related species within taxa by employing housekeeping or fungal effector genes [16,18,19].

Housekeeping genes, as slowly evolving regions that encode proteins essential for microorganism survival [20], are suitable to investigate the evolutionary pathways of taxa emergence and the relations between them, e.g., for species delimitation, and to investigate genetic relatedness among strains and populations of Eukaryota taxa, including plant or human pathogenic fungi [21,22,23], while, at the same time, conserved gene markers may exhibit insufficient intraspecies genetic variation to differentiate strains [22]. Fungal effector genes are genes coding molecules involved in interactions with host plants, but are not necessary for a pathogen to complete its lifecycle in vitro, such as those encoding infection structures, CWDEs, toxins or components suppressing the plant immune response; their products can act as elicitors or virulence and avirulence factors [24,25,26]. In MLSA of the putative effector genes, it is possible to utilize patterns of nucleotide diversity to infer the molecular base of pathogen coevolution with its host plant [27]. This approach implies that regions connected to pathogenicity specialized to host cultivars are under selective pressure exerted by host resistance genes and are expected to differ between pathogenic races or types [28]. An adaptation to the new ecological niches depends mostly on the genes located on the variable regions of the genome, which can adapt more quickly than the conservative parts of the genome, thereby causing detectable differences between them. To the best of our knowledge, these regions have never been investigated in the context of phylogenetic analyses in *V. inaequalis* or in terms of their diversity in relation to the virulence abilities of *V. inaequalis* races.

In this study, two housekeeping genes, elongation factor alpha (EF-1α) and β-tubulin, and two previously unstudied effector genes of *V. inaequalis*, one from mannosidase and one from the glucosidase family, were investigated to infer phylogenetic relationships and genetic structure within the species and to detect whether there is any relationship between genetic variation and host specialization among the strains of *V. inaequalis* species.

The aims of the study were to assess (a) the phylogenetic structure of the pathogen within the species to answer the question of whether the populations that are virulent or avirulent to *Rvi6* cultivars belong to distinct phylogenetic lineages; (b) the genetic divergence among groups of strains to identify intraspecific populations; (c) the evolutionary forces that are currently acting on this microorganism; and (d) the possible occurrence of recombination events within the species. To investigate these aims, we applied phylogenetic, diversity and recombination analyses to the sequence data of four genetic loci.

## 2. Results

### 2.1. Phylogenetic Analyses

As a result of the amplification of gene fragments of 100 strains of *V. inaequalis* (Table 1), products of the following sizes were obtained: 436 or 487 bp for β-tubulin, 385 bp for EF-1α, 858 bp for mannosidase and 1006 bp for glucosidase, while the length of the sequences after trimming for final alignments was slightly shorter (as listed in Table 2a).

The partition homogeneity test for housekeeping and pathogenicity-related genes demonstrated that combining the data generally improved phylogenetic accuracy (*p* > 0.01, ref. [29]); thus, the nucleotide sequences of the genes could be concatenated.

**Figure 1:**
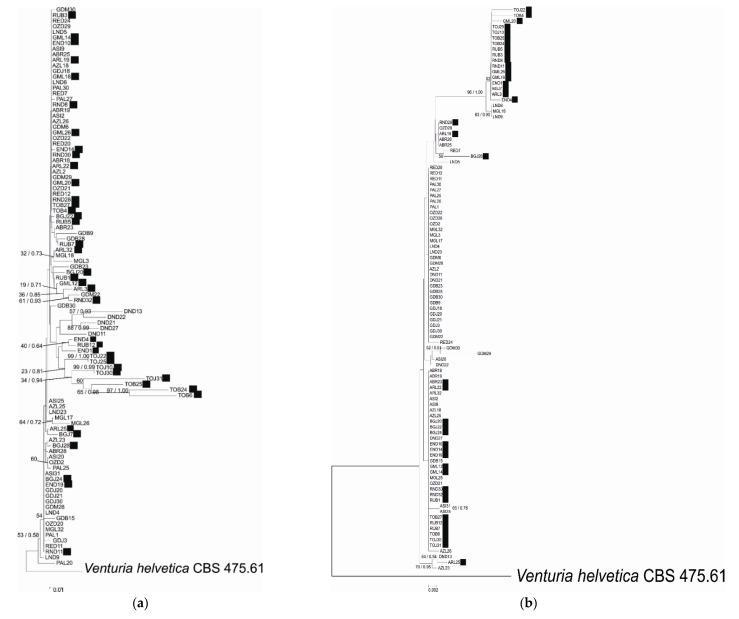

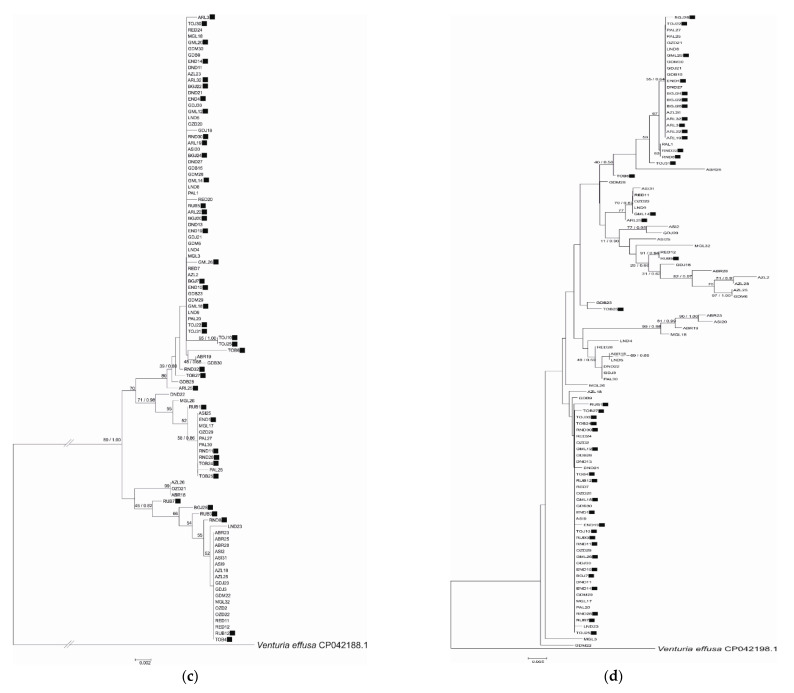

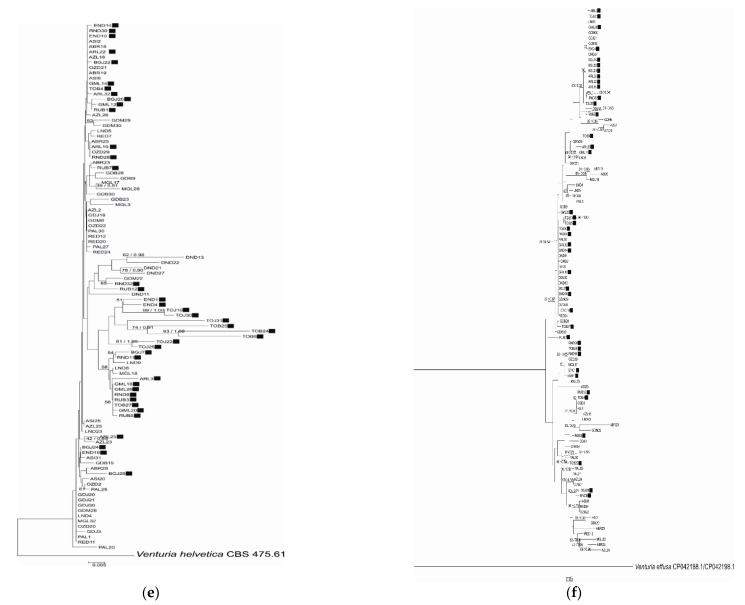
Phylogenetic relationship of *Venturia inaequalis* based on Neighbor Joining analysis of (**a**) EF-1α, (**b**) β-tubulin, (**c**) mannosidase and (**d**) glucosidase sequences and concatenated data: (**e**) EF-1α and β-tubulin and (**f**) mannosidase and glucosidase. Bayesian posterior probabilities values (Bpp) are given for common nodes next to NJ bootstrap values (1000 replicates), which are indicated close to the nodes. Bootstrap values above 50% are given. The scale bars represent the number of base substitutions per site. Black squares indicate *Rvi6*-virulent strains.

Although slightly different relationships between leaves among dendrograms obtained using Maximum Parsimony, Maximum Likelihood and Neighbor-Joining) criteria were observed, the genealogy of each locus was best resolved (visualized) by a single rooted NJ tree. The topologies of the trees representing all the regions and combined datasets were similar in the NJ and Bayesian analyses; therefore, only NJ trees are presented (Figure 1a–f), with bootstrap values being shown for well-supported branches and Bpp values being given for co-occurring nodes. Each tree had a minimal length equal to the number of polymorphic sites in that locus.

The phylogenetic analysis of EF-1α, β-tubulin, mannosidase and glucosidase regions or combined datasets of the housekeeping- or pathogenicity-related genes indicated slightly different topologies between the studied gene fragments. None of the genealogies resolved the isolates from different cultivars as distinct clades according to virulence type towards *Rvi6*, as was expected from previous studies. Moreover, no relation between strain position and its origin, i.e., plant cultivar or geographic localization, was detected. The visual inspection of well-supported nodes exhibited no particular topological congruence among individual gene trees. The majority of nodes on trees were supported with low bootstrap values (below 50), indicating low variation among strains.

### 2.2. Intragene Polymorphisms

The lowest average sequence similarity within the analyzed fragment was observed for EF-1α (93.3%), while the highest was observed for mannosidase (98.9%). The G+C content was similar for all analyzed regions and ranged from 0.481 to 0.569, and the lowest G+C content was observed in mannosidase (below 50%). The highest percentage of parsimony informative sites and segregating sites (S), as well as the number of haplotypes per locus, were counted for EF-1α, although this gene fragment was the shortest one analyzed. Similarly, gene diversity (Hd) and Theta-W values were the highest for this region.

Only for EF-1α was the null hypothesis of neutrality rejected in both the Tajima’s D and the Fu and Li’s D tests. A significant negative value of D, but in only one of the tests, Fu and Li’s D test, was also observed for the β-tubulin gene. Positive or negative values of D statistics, although not significant, were observed for mannosidase and glucosidase, indicating that these regions are evolving close to mutation–drift equilibrium, and no evidence of selection can be observed. In concatenated dataset analysis, the number of parsimony informative sites, the percentage of segregating sites (S) sites and haplotypes as well as two parameters of nucleotide diversity (π and Theta W) and gene diversity (Hd) were higher for combined housekeeping genes than for pathogenicity-related genes (Table 2b). Only for concatenated EF-1α and β-tubulin genes were the values of Tajima’s D and Fu and Li’s D significantly negative. When *Rvi6*-virulent and -avirulent populations were compared for single loci, the percentage of segregating sites (S) and Theta-W values were higher in *Rvi6* populations, with the exception of glucosidase (Table 2c). A value of nucleotide diversity (π) was higher in *Rvi6*-virulent populations for EF-1α and β-tubulin, while it was higher in *Rvi6*-avirulent populations for mannosidase and glucosidase regions. The percentages of haplotypes and parsimony informative sites were higher in *Rvi6*-avirulent populations for β-tubulin and glucosidase regions, while these values were higher in *Rvi6*-virulent populations for EF-1α and mannosidase regions, which corresponded to the distribution of the gene diversity (Hd) values for these regions. For EF-1α, β-tubulin and glucosidase, values of Tajima’s D test were higher for *Rvi6* populations, although only for EF-1α were they significant.

The results of the McDonald–Kreitman test, conducted on coding sequences, revealed that in EF-1α and glucosidase gene fragments, there is a twofold increased number of non-neutral polymorphisms compared to the number of neutral polymorphisms (Table 3), while in the β-tubulin region, the same number of neutral and non-neutral polymorphisms was detected. In mannosidase coding regions, the number of synonymous polymorphisms was almost nine times higher than the number of non-synonymous polymorphisms, which was congruent with the topology of the dendrogram constructed based on the protein sequence: on the tree obtained for mannosidase, the smallest number of leaves was observed among all obtained phylograms, both nucleotide and protein based (data not shown). The highest diversity in protein sequence (the largest number of leaves) was observed on the protein dendrogram obtained for the glucosidase coding region.

### 2.3. DNA Divergence and Differentiation between Strains of Rvi6-Virulent and -Avirulent Populations

Gene flow estimates of Nei [30], represented by Nm values calculated in DnaSP between *Rvi6*-virulent and -avirulent populations, were highest for EF-1α and lowest for β-tubulin (Table 4). Additionally, the average number of nucleotide substitutions per site between these populations (average pairwise divergence, D_XY_) was the highest for EF-1α, while the average number of nucleotide differentiations between populations was the highest for glucosidase.

Analysis of molecular variance (AMOVA) among the two groups, that is, one containing only *Rvi6*-virulent populations and the other containing *Rvi6*-avirulent populations, demonstrated that the largest genetic variation was partitioned between individuals within populations (65.6–91.7%, depending on the gene), while the lowest values were observed among groups (0.6–3.2%) (Table 5). The exception was observed for the β-tubulin gene, for which the observed variation among groups was 31.9%, while the lowest variation was observed among populations within groups (2.5%). When each group of populations was considered separately for each gene (only *Rvi6*-virulent or *Rvi6*-avirulent populations in the group), the greatest genetic variation was again partitioned between individuals within populations (from 77.0 to 96.6%), regardless of the gene and group, but the variation among populations was significantly higher in the *Rvi6*-virulent group than in the *Rvi6*-avirulent group for the analyzed genes with the exception of the β-tubulin region. In this instance, variation among populations was higher for the *Rvi6*-avirulent group than for the *Rvi6*-virulent group.

Compared between the *Rvi6*-virulent and -avirulent populations, significant F_ST_ values were higher for the two analyzed housekeeping genes, 0.34 and 0.23 for β-tubulin and EF-1α, respectively, than the values obtained for genes related to pathogenicity: 0.16 and 0.08 for mannosidase and glucosidase, respectively (Table 5). This result indicates that genetic distance (restricted gene flow and genetic isolation between the two populations) inferred from housekeeping genes is higher among the two population types, while the higher similarity of genes related to pathogenicity results from possible gene flow between strains within these regions.

When *Rvi6*-virulent and *Rvi6*-avirulent population groups were compared for each region, a closer relationship was usually observed among *Rvi6*-avirulent populations (for EF-1α, mannosidase and glucosidase regions), while *Rvi6*-virulent populations were more distanced (Table 5). Again, an exception was observed for the β-tubulin region: gene flow related to the F_ST_ value was higher among *Rvi6*-virulent populations than among *Rvi6*-avirulent populations.

### 2.4. Recombination and Linkage Disequilibrium Analysis

The extent of linkage disequilibrium and recombination between and within *Rvi6*-virulent and -avirulent populations was assessed using a) split decomposition and recombination detection analyses, b) index of association (rBARd) and c) the four gametes test for the detection of the minimum number of recombination events (*R_M_*).

(a) Split decomposition analyses revealed a lack of reticulation among strains for all studied regions analyzed as separate or concatenated datasets, suggesting a lack of recombination between strains. However, the phi test did find statistically significant evidence for recombination (*p* < 0.05) in glucosidase and mannosidase genes but not in EF-1α and β-tubulin loci. Additionally, recombination signals were detected using two out of seven algorithms under RDP4 for the glucosidase locus only. Consequently, evidence for recombination in the phi test, as well as recombination signals, were detected in the concatenated mannosidase and glucosidase dataset only (in three of seven used algorithms under RDP4).

(b) The random recombination hypothesis was tested both for all strains without any grouping assumptions, as well as for the strains divided into two assumed populations: *Rvi6*-virulent and -avirulent. In both analyses, rBARd values were significantly higher than 0 (*p* < 0.01) for all analyzed regions and combined pathogenicity-related regions (Table 6), showing that the null hypothesis that assumes a lack of association could be rejected for all strains and between the strains and groups of strains for which recombination is restricted. This phenomenon was observed for all regions, which indicates that all analyzed regions showed evidence for linkage disequilibrium. The highest index of multilocus association was observed for mannosidase (0.5005). However, random recombination was possible when concatenated datasets of EF-1α and β-tubulin were analyzed—either among all strains or for the assumed two populations (*p* > 0.05). Complete panmixia, related to rBARd values near 0, is expected for fully recombining populations, while in linkage disequilibrium, a deviation from panmixia is observed, related to an rBARd value significantly greater than 0.

(c) Data were considered to have failed the simple four gamete test if the minimum number of recombination events (*R_M_*) needed to explain the alleles was ≥1 [31]. Estimation of intralocus recombination parameters showed that the minimum number of recombination events ranged from three to 20 across loci; this value was the highest for the EF-1α gene among single loci (Table 4). Interlocus recombination events equaled 23 and 26 for combined datasets of housekeeping- and pathogenicity-related genes, respectively.

## 3. Discussion

The advantages of DNA sequencing and advances in phylogenetic analysis have provided a powerful means of studying the differentiation and evolution of fungal species. Using multilocus sequence analyses, it is possible to detect evolutionary processes at the strain, population and species levels, leading to differentiation.

### 3.1. Phylogenetic Relationships between Rvi6-Virulent and Rvi6-Avirulent Strains

The housekeeping genes used in the study, EF-1α and β-tubulin, are frequently used as molecular phylogenetic markers to deduce the evolutionary history and genetic variation of morphologically similar fungal species [32] and also reliably estimate the phylogenetic relationships within *Venturia* [33]. In our study, although the EF-1α gene showed the highest phylogenetic signal from the analyzed regions (the highest percentage of parsimony informative and segregating sites) and the concatenated dataset based on housekeeping genes had more phylogenetic signals that concatenated pathogenicity-related regions, it was not possible to differentiate the *V. inaequalis Rvi6*-virulent from -avirulent strains based on these regions in our study, which can be explained by insufficient variation in housekeeping gene sequences. Although multiple gene sequences are evolving with different substitution patterns, concatenating gene sequences into a supergene alignment may provide more accurate trees [33]; however, in our study, concatenated data did not exhibit discrimination between *Rvi6*-virulent and -avirulent populations. All strains used in this study, representing four pathogenic races (according to Bus et al., 2011 [2]), were examined previously by multilocus microsatellite analysis [11]. These and other reports, comprising *V. inaequalis* populations from European orchards, demonstrated that pathogen populations, sampled from various apple tree cultivars, are divided into two main groups, where belonging to a group is conditioned by virulence or avirulence towards the *Rvi6* host gene [5,9,10,11]. Therefore, our experiment aimed to verify whether this division is also detectable in genic regions, especially those potentially related to pathogenicity. Our results indicated no correlation between the genetic features of the tested strains and their geographical origin, host cultivar or pathological race. Similarly, Zhao et al. [33] reported that intraspecific phylogenetic relationships among strains belonging to different pathological races in *V. nashicola* were not detected. Additionally, Gladieux et al. [7] observed that none of the single-locus genealogies showed a clear partitioning of isolates from various host plants into separate clusters; however, they observed a substantial level of differentiation among three groups: infecting loquat, pyracantha or apple, indicating that some forces act to counteract free exchanges of genetic material. In this instance, isolates from different hosts were differentiated but did not form diagnosable distinct phylogenetic groups. Moreover, many clades or subclades were resolved with low (below 50%) bootstrap values, indicating that overall sequence diversity is not high. This phenomenon may be observed when very conserved DNA fragments are used and sequences are too similar to each other; thus, the number of informative sites is too small, and variation is too low, and grouping into branches is therefore equivocal.

### 3.2. Factors Influencing the Genetic Diversity and Divergence among Rvi6-Virulent and Rvi6-Avirulent Strains

Although it was reported that sequences of genes encoding pathogenicity factors, such as endopolygalacturonase and exopolygalacturonase, have been used and proved to be helpful to reveal the relationship of genetic divergence with pathogenicity variation [28], in our study, this relation was undetectable for strains showing different virulence patterns (i.e., virulence to different *Rvi6* hosts) based on pathogenicity-related regions. In contrast to microsatellite markers [11], both housekeeping- and pathogenicity-related genes selected in this study were insufficient in separating strains of known virulence patterns in this study. The possible reason for this finding is that analyzed genes are not connected with the genome regions related to pathogen virulence to various cultivars of plant hosts or are located in the conservative regions of the genome; thus, they do not adequately reflect the genetic diversity within the taxon for the purpose of race discrimination. Another possibility is that microsatellites, which are randomly scattered beyond genic regions of the genome, and are highly polymorphic and variable DNA structures, have much higher mutation rates operating at the microsatellite loci than do point mutations in genic regions [34], which leads to greater intrapopulation genetic diversity; thus, any traces of genome rearrangements should be first detected in microsatellite regions at the population level.

To demonstrate the genetic diversity, population divergence and neutrality of mutations, we used Tajima’s D and Fu and Li’s D tests. Values of Tajima’s D test were higher in *Rvi6* populations for three of four analyzed loci, while in *Rvi6*-avirulent populations, they were higher for mannosidase. Tajima’s D may be higher in populations that have experienced a recent bottleneck because of the preferential loss of low-frequency variants [35]. The expected pattern of average Tajima’s D values through time after a bottleneck includes an initial period of positive D values, declining towards a period of negative D values, and finally turning back towards equilibrium [36]. However, the values for only one gene, EF-1α, were significantly negative, which may be related to a recent selective sweep, linkage of the analyzed region to a swept gene or population expansion after a recent bottleneck, while the rest of the regions are under neutral selection. Moreover, the values of gene flow (expressed as Nm, DnaSP) and average pairwise differentiation (Dxy) calculated between virulent and avirulent *Rvi6* strains were the highest for the EF-1α gene and the lowest for β-tubulin. This finding may suggest that housekeeping genes may be under selective pressure to retain function, although the pressure can act on some regions only. In addition, the McDonald–Kreitman test conducted between the two groups of strains demonstrated diversifying selection acting on EF-1α and glucosidase gene fragments, while the β-tubulin region was under neutral selection, and the mannosidase protein fragment was the most evolutionarily conserved in the study. More detailed studies are necessary to establish which genes are relevant for the adaptation of *V. inaequalis* strains to the defined virulence type.

Moreover, in most cases (that is, for three of four loci), higher variation was observed among *Rvi6*-virulent populations than in *Rvi6*-avirulent populations, which was congruent with gene flow F_ST_ estimates, indicating that populations in the *Rvi6*-virulent group were more distanced, while those in the *Rvi6*-avirulent group were closely related. Meanwhile, higher gene diversity was observed for two regions in *Rvi6*-virulent populations, while for two other regions, it was observed in *Rvi6*-avirulent populations. Usually, a higher number of segregating sites, nucleotide diversity and Theta-W values were found in *Rvi6*-virulent populations than in *Rvi6*-avirulent populations for three loci. Selection might not be the main reason for the severe reduction of diversity in *Rvi6*-avirulent populations, although the effects of selection cannot be excluded entirely. The reduced level of genetic diversity may reflect the consequence of evolutionary processes, such as a recent speciation event, population bottleneck or selective sweep for a preferred genotypic variant in *V. inaequalis* [37]. This finding is in contrast to the results obtained in multilocus microsatellite loci, where *Rvi6*-virulent populations were characterized by low gene diversity, even demonstrating a clonal structure [6,8,11]. It has been proven that *Rvi6* populations are the descendants of a few genetically different virulent individuals that migrated relatively recently to Poland from non-agricultural reservoirs, and they are not abundantly represented in our county at present. In contrast, *Rvi6*-avirulent populations, commonly occurring in our agrosystem for decades and which are evolutionarily older, were characterized by higher allelic and private allelic richness, as well as higher genetic diversity [11]. Our current finding about the level of diversity of the studied strains, based on gene regions, may suggest that the examined *Rvi6*-avirulent individuals are only a small representation of the whole *Rvi6*-avirulent population in Poland and that the rate and mechanism of evolution are different for genic and microsatellite regions, and their effects cannot be compared. These genes were studied to assess another level of genetic variation between pathogen populations. However, the examined regions are not sufficient to resolve differentiation among *Rvi6*-virulent and -avirulent populations, while generally, genetic diversity between populations is supposed to be a factor facilitating adaptation of pathogens to specific resistance genes of the host both by increasing risks of avirulence losses and the spread of new virulences.

### 3.3. Recombination between Rvi6-Virulent and Rvi6-Avirulent Strains

Depending on the algorithm used, only in glucosidase or in the glucosidase and mannosidase concatenated dataset were recombination signals detected. On the other hand, estimation of the index of association based on four single loci demonstrated that random mating was restricted both among all strains and between assumed groups of strains, that is, *Rvi6*-virulent and -avirulent, which indicates that some loci can be associated. Theoretically, linkage disequilibrium is affected by various evolutionary and demographic factors, including selection, recombination, population admixture, inbreeding and bottlenecks [38]. The restricted recombination between the strains and between assumed groups is consistent with a model of reproductively isolated populations (i.e., virulent or avirulent towards *Rvi6* cultivars). However, this reproductive barrier or significant genetic differentiation between groups cannot be explained by geographic isolation, since strains of different virulence were sampled in the same orchard in half of the cases (strains from Lublin and Nowy Dwor). Based on the data obtained in this study, we did not find evidence of gene flow after secondary contact in the analyzed regions, as was previously observed for virulent and avirulent *Rvi6* populations based on microsatellite markers [11]. Moreover, in the absence of recombination, all gene trees should be congruent, and alleles at different gene regions should be associated [17]. In our study, visual comparison of the dendrograms constructed based on four loci and concatenated data showed no compatibility between them; strains from various races were scattered randomly without a clear tendency in grouping and independent of the analyzed region. Moreover, the association of alleles was detected for groups of strains. These data confirm the presence of recombination between strains and enable us to assume that the analyzed regions are in linkage disequilibrium and contain non-random polymorphisms associated with the strain.

## 4. Materials and Methods

### 4.1. Collection of Fungal Samples

During the late spring and early summer of 2012–2014, infected leaves with clear scab lesions were randomly collected in six chemically controlled apple tree orchards, two organic fungicide-free orchards and two orchards employing mixed types of control (i.e., containing both non-chemically and chemically protected cultivars) located in the central, southern and northern parts of Poland. In all the orchard trees of *Malus* x *domestica*, in addition to one location where leaves of crab apple trees were obtained, F1 seedlings of Malus x zumi var. “Colocarpa” originating from open pollination (OZD, Dąbrowice) were collected. The sampled apple trees represented both mono-R-genic cultivars (*Rvi1*, *Rvi6* or *Rvi17*) and cultivars without any known sources of apple scab resistance (hereafter designated Rvi0). The scab lesions were sampled and prepared for the analysis as described previously [11]. In total, DNA was obtained from 100 strains of *V. inaequalis*, which were divided into 20 groups according to cultivar and/or location, each containing five strains, and the DNA was subjected to further analyses (Table 1).

### 4.2. Designing Primers for PCR and Amplification of Pathogenicity-Related Genes

The genome of *V. inaequalis* (“Venin1”), provided by Cecilia Deng at the New Zealand Institute for Plant and Food Research Limited, available at https://genome.jgi.doe.gov/Venin1/Venin1.home.html (accessed on 3 February 2021), was searched for CWDE sequences. Among these sequences, two contigs, one complementary to mannosidase and one to glucosidase of Venin1, were selected, and primers were designed for them using PrimerSelect software of the Lasergene v. 8 package (DNASTAR, Madison, WI, USA). The specificity of designed primer sets was tested in conventional PCRs using DNA from 100 isolates of *V. inaequalis* pure culture (collection of Research Institute of Horticulture, Skierniewice, Poland). After the optimization process was undertaken, two primer sets were selected and used for the amplification of part of mannosidase and glucosidase gene sequences, VinManno1 and VinManno2, and VinGluco1 and VinGluco2 (Table 7), respectively, in PCR with DNA of all strains collected in the study (Table 1). The conditions for each reaction consisted of an initial denaturation at 95 °C for 3 min followed by 35 cycles of denaturation at 95 °C for 30 s, annealing at 61 °C for the primers VinManno1 and VinManno2 or at 60 °C for the primers VinGluco1/VinGluco2 for 30 s, polymerization at 72 °C for 60 s and a final extension step at 72 °C for 5 min. Each reaction was performed in a total volume of 30 µL containing 1–10 ng of DNA, 0.133 mM of each dNTP, 500 nM of each primer, 0.9 U of DreamTaq DNA Polymerase (Thermo Fisher Scientific, Vilnius, Lithuania), 1 × optimized DreamTaq Green Buffer (Thermo Fisher Scientific, Vilnius, Lithuania) and double-distilled water.

### 4.3. Amplification of Housekeeping Genes

For the sequence analyses of housekeeping genes of all studied strains, two genes were selected, encoding EF-1α and β-tubulin. A fragment of DNA of EF-1α was amplified using the VNEFI-f/VNEFI-r primer set [33], while the fragment of the β-tubulin gene was amplified with primers C/D [39]. For 16 strains, no product was obtained by PCR with the C/D primer set; therefore, new primers were designed. The new primers cont189 and cont675 (Table 3) were complementary to DNA regions of the β-tubulin gene and were located upstream and downstream in relation to the C/D primer annealing sites. Thermal profiles of reactions and reagent concentrations applied for PCR with the primers used for housekeeping genes were the same as those applied for pathogenicity-related genes with the exception of annealing temperatures: 61 °C for VNEFI-f/VNEFI-r and C/D primer sets and 59 °C for cont189/cont675 primer set.

### 4.4. Polymorphism and Intragene Differences Analyses

The products obtained in all PCRs were separated in a 1.5% agarose gel in Tris-borate-EDTA buffer, stained with ethidium bromide, and visualized under UV illumination; next, the products were purified using a PCR/DNA Clean-Up Purification Kit (EURx, Gdańsk, Poland) and sequenced in both directions with the same primers used for amplification (Genomed SA., Warsaw, Poland). The obtained sequences for each gene fragment were assembled and initially aligned using MEGA v. 6.0 software ([40]; available at www.megasoftware.net/mega.php (accessed on 3 February 2021)). Alignment was then visually refined, and the consensus sequence for each gene was employed as a query in Nucleotide Basic Local Alignment Search Tool (BLAST) to search the global GenBank sequence database, available at https://blast.ncbi.nlm.nih.gov/Blast.cgi (accessed on 3 February 2021), and the *V. inaequalis* reference genome sequence. Alignments per gene with all sequences trimmed to the same length, as well as concatenated alignments of housekeeping or pathogenicity-related genes, were exported to nexus files. All sequences used in the study were submitted to the GenBank database, and accession numbers were assigned for them (Supplementary File 1).

A partition homogeneity test was performed in Phylogenetic Analysis Using Parsimony (PAUP) version 4.0b10 [41] to ensure that the two housekeeping or two pathogenicity-related genes could be combined to form a single concatenated dataset [29].

The percentage of similarity between sequences was calculated for each gene fragment using MegAlign software from the Lasergene v. 8 package.

For each gene, the number of haplotypes (h), number of polymorphic (segregating) and parsimony informative sites, G/C content and haplotypic diversity (Hd) were calculated, and 2 parameters of nucleotide diversity, polymorphism (π—the expected heterozygosity per nucleotide site [42], or average pairwise divergence between isolates) and Theta-W (dependent on effective population size and mutation rate per nucleotide), were estimated using DnaSP v. 5.10.0.1 [43]. The same parameters were also estimated for sequentially end-to-end concatenated sequences of EF-1α with β-tubulin and mannosidase with glucosidase regions.

When two main groups of populations were assumed, that is, *Rvi6*-virulent and -avirulent, gene flow estimates between groups (Nm and Gst), the average number of nucleotide differentiations between groups and the average number of nucleotide substitutions per site (average pairwise divergence between populations, Dxy) were calculated.

Tajima’s D genetic diversity and population divergence test [44] and Fu and Li’s D tests for neutrality of mutations [45] were also carried out. For both tests, negative values indicate an excess of low-frequency polymorphisms, e.g., rare substitutions are present at higher frequencies in the region than expected under a standard neutral model [46], while positive values indicate an excess of intermediate polymorphisms. If the value of D is too large or too small, the neutral null hypothesis is rejected, and population bottlenecks or population subdivisions, as well as selective sweeps, are allowed [47]. Both tests were assessed for their statistical significance according to Kimura neutral theory [48]. In addition, the McDonald–Kreitman test of neutrality (at http://bioinf3.uab.cat/mkt/MKT.asp, accessed on 3 February 2021) was applied to coding sequences of the studied gene fragments to provide evidence of selection in the case of non-neutral polymorphism [47]. The test calculated the ratio between synonymous and non-synonymous polymorphisms between *Rvi6* and non-*Rvi6* populations of *V. inaequalis*. All nucleotide sequences were converted to amino acid sequences according to an adequate reading frame using the EXPASY translate tool (https://web.expasy.org/translate/, accessed on 3 February 2021) and were subsequently utilized to construct neighbor-joining dendrograms (NJ, MEGA6) to visualize the topologies of amino acid sequence-derived trees with DNA sequence-derived trees.

### 4.5. Structure Analyses

Gene flow estimates of Nei [30], the average number of nucleotide substitutions per site between these populations (D_XY_) and the average number of nucleotide differentiations between populations were calculated between assumed populations (*Rvi6*-virulent vs. *Rvi6*-avirulent) using the DnaSP program.

The gene flow estimates between *Rvi6*-virulent and -avirulent populations were also assessed based on the fixation index (F_ST_) measuring the population differentiation among strains, applying 100 permutations using ARLEQUIN 3.5.1.2 [49]. Estimation of gene flow, expressed as F_ST_, can range from 0 (no differentiation between populations due to unrestricted gene flow) to 1 (complete isolation due to the absence of gene flow) [50]. Each of the 4 genes was used independently for analyses. For AMOVA purposes, all strains were divided into 20 populations, with each containing 5 strains according to their origin, and they were subsequently included in two main assumed groups of populations: one containing only *Rvi6*-virulent populations and the other containing only *Rvi6*-avirulent populations. The total amount of differentiation among the groups of populations, among populations within the groups and individuals within populations was evaluated by applying global hierarchical analysis of molecular variance (AMOVA) using 1023 permutations and a significance level of *p* ≤ 1 for single loci.

### 4.6. Model Selection and Phylogenetic Analysis

The best fit evolutionary model of DNA substitution for four datasets was calculated using jModelTest v. 2.1.1 [51]. The standard Akaike information criterion (AIC) was used to evaluate the model scores. The appropriate evolution model for β-tubulin was indicated as JC+I+G (combined: Jukes–Cantor with a proportion of invariable sites and a gamma-shaped distribution of rates across sites) for EF-1α, JC+I for β-tubulin, K80+I (combined: Kimura two-parameter with a proportion of invariable sites) for mannosidase and HKY+I+G (combined: Hasegawa–Kishino–Yano model with a proportion of invariable sites and a gamma-shaped distribution of rates across sites) for glucosidase; JC+I+G for combined datasets of housekeeping genes and GTR+I+G (general time-reversible model with a proportion of invariable sites and a gamma-shaped distribution of rates across sites) for combined datasets of pathogenicity-related genes. The optimal models of nucleotide substitution, as determined by jModeltest, were used for the distance-based approaches and phylogenetic tree reconstruction.

Phylogenetic analyses were performed by a heuristic search for optimal trees under three optimality criteria: parsimony (MP), likelihood (ML) and distance (NJ) using MEGA6. NJ trees were constructed based on model-based corrected distances, as indicated by jModelTest for each dataset.

The strength of the internal branches of the resulting trees was tested with bootstrap analyses using 1000 replications in MEGA6.

MrBayes v. 3.2.6 [52] was used to calculate the Bayesian posterior probability values (Bpp) for each gene region and the concatenated EF-1α with β-tubulin and mannosidase with glucosidase datasets. Parameters and priors were modified to reflect the evolutionary model that best represented the data according to jModelTest; next, metropolis-coupled Markov chain Monte Carlo (MCMCMC) analysis was performed with 2 independent internal runs for 5 × 106 generations and burn-in of 25% generations with 4 chains. All analyses were performed twice to ensure that they were not trapped at local maxima, and the overall tree topologies were compared to ensure that the analyses converged on a similar phylogeny.

### 4.7. Detection of Recombination

The split decomposition method [53] was used to visualize incompatibilities generated by recombination using SplitsTree4 software, and the nucleotide substitution model indicated the best fit for the given dataset. Bootstrapping values for the split decomposition network were calculated with 1000 replicates with SplitsTree4 software [54]. For the detection of recombination or genomic reassortment signals in analyzed regions, the phi test for recombination under SplitsTree4 and seven different recombination detection methods implemented in RDP4 software [55] were employed: RDP, Geneconv, Bootscan, MaxChi, Chimaera, SiScan and 3 Seq.

Association among alleles, as expected under clonality, was investigated using a generalized measure of multilocus linkage equilibrium (rBARd) [56,57], which is a modification of the index of association (IA) and is independent from the number of loci included in the analysis. Each gene and two combined datasets were analyzed using MULTILOCUS v. 1.2.2. software [57]. Values were calculated for full datasets, arranged in two variants: (i) when all strains were treated as one population and (ii) when two main populations were assumed, that is, one containing strains derived from *Rvi6* cultivars and one containing the rest of the strains. The significance of the test was assessed by comparing the original observed dataset with those in which alleles were resampled without replacement to simulate recombination in 100 randomized datasets.

Finally, the simple four gamete test was performed within each locus and combined dataset using DnaSP to estimate the minimum number of recombination events in the history of each gene sample (*R_M_*).

## 5. Conclusions

The four loci examined in this study are only a small part of the total genome. In our study, the total information obtained from these data did not demonstrate one consensus phylogenetic resolution for the analyzed strains; probably, involvement of several additional regions would be required to obtain more informative phylogenetic trees. On the other hand, these data provided some indications related to evolutionary forces affecting the strains belonging to different pathological races, either virulent or avirulent to *Rvi6* host plants, and these results confirm higher genetic distance and variation among *Rvi6*-virulent populations than among avirulent ones and are in contrast to some results from previous studies based on microsatellites concerning the level of genetic diversity in *Rvi6*-virulent and -avirulent populations. Discrimination between *V. inaequalis* populations of different virulence types is achieved primarily by testing their pathogenicity on apple cultivars with defined R genes [2]; nevertheless, it is better achieved by multilocus microsatellite genotyping than by multilocus gene analysis presented in this report, indicating that the latter cannot serve as markers for molecular discrimination among *V. inaequalis* races.

## Figures and Tables

**Table 1:** The names and origins of the *V. inaequalis* populations.

R Gene of Host	Orchard Location, Cultivar	Control Type	Population Names	No. of Strains Used
0	Dabrowice, F1 seedling of *Malus* x *zumi* var. “Colocarpa”	organic	OZD	5
0	Lublin, Gala	organic	MGL	5
0	Lublin, Paulared	organic	PAL	5
0	Nowy Dwor, Ligolina	organic	LND	5
0	Nowy Dwor, Delbard Jubile	organic	DND	5
*Rvi1*	Brzezna, Golden Delicious	chemical	GDB	5
*Rvi1*	Jajkowice, Golden Delicious	chemical	GDJ	5
*Rvi1*	Milobadz, Golden Delicious	chemical	GDM	5
*Rvi6*	Nowy Dwor, Enterprise	organic	END	5
*Rvi6*	Nowy Dwor, Rajka	organic	RND	5
*Rvi6*	Brzeziny, Rubinola	organic	RUB	5
*Rvi6*	Brzeziny, Topaz	organic	TOB	5
*Rvi6*	Jeziorsko, Topaz	organic	TOJ	5
*Rvi6*	Jeziorsko, Biogolden	organic	BGJ	5
*Rvi6*	Lublin, Ariwa	organic	ARL	5
*Rvi6*	Lublin, Gold Milenium	organic	GML	5
*Rvi17*	Brzezna, Antonovka	organic	ABR	5
*Rvi17*	Siedlce, Antonovka	organic	ASI	5

**Table 2:** Parameters of sequence polymorphism, nucleotide diversity and neutrality tests for single loci (**a**), concatenated data (**b**) and estimated for assumed groups of populations of *V. inaequalis*: *Rvi6*-virulent and *Rvi6*-avirulent (**c**).

(**a**)												
**Gene Fragment**	**Length in Basepairs**	**G/C Content**	**No. of Polymorphic/Segregating Sites [S]**	**Polymorphic Sites in %**	**% of Haplotypes Per locus**	**No. of Parsimony Informative Sites**	**Haplotype [Gene] Diversity [*Hd*]**	**Nucleotide Diversity [π]**	**Theta per Site from S [** **θ** **-W]**	**Tajima’s Test D**	**Fu and Li’s D Test for Neutrality of Mutations**	**Average Percentage of Sequence Similarity between Strains**
EF-1α	336	0.505	103	30.7	49	64	0.869	0.02109	0.05921	−2.29955 *	−2.58706 *	93.3
β-tubulin	384	0.529	23	6	22	12	0.800	0.00813	0.01160	−1.13774	−2.92669 *	98.45
mannosidase	784	0.481	27	3.4	25	21	0.741	0.00994	0.00665	1.33244	−0.18782	98.9
glucosidase	533	0.569	66	12.38	41	49	0.862	0.02321	0.02514	−0.42071	−0.90093	95.8
(**b**)												
**Gene fragment**	**Length in basepairs**	**G/C content**	**No. of polymorphic/segregating sites [S]**	**Polymorphic sites in %**	**% of haplotypes per population**	**No. of parsimony informative sites**	**Haplotype [gene] diversity [*Hd*]**	**Nucleotide diversity [π]**	**Theta per site from S [** **θ** **-W]**	**Tajima’s test D**	**Fu and Li’s D test for neutrality of mutations**	
all strains, concatened EF-1α and β-tubulin	720	0.518	126	17.5	67	76	0.974	0.1416	0.03385	−2.12766 *	−2.95196 *	
all strains, concatened mannosidase and glucosidase	1317	0.556	93	7.06	61	70	0.955	0.01515	0.01466	0.1097	−0.74098	
(**c**)												
**Gene fragment**	**Length in basepairs**	**G/C content**	**No. of polymorphic/segregating sites [S]**	**Polymorphic sites in %**	**% of haplotypes per population**	**No. of parsimony informative sites**	**Haplotype [gene] diversity [*Hd*]**	**Nucleotide diversity [π]**	**Theta per site from S [** **θ** **-W]**	**Tajima’s test D**	**Fu and Li’s D test for neutrality of mutations**	
EF-1α *Rvi6*-virulent	336	0.504	75	22.3	60	46	0.89359	0.02859	0.05248	−1.87014 *	−1.25818	
EF-1α *Rvi6*-avirulent	336	0.505	64	19	47	27	0.85367	0.01530	0.04049	−2.24047 *	−3.11016 *	
β-tubulin *Rvi6*-virulent	384	0.528	18	4.7	22.5	7	0.67949	0.00957	0.01105	−0.43613	−2.49531	
β-tubulin *Rvi6*-avirulent	384	0.529	17	4.43	25	11	0.70226	0.00467	0.00952	−1.72921	−1.15951	
mannosidase *Rvi6*-virulent	784	0.48	24	3.06	35	20	0.72740	0.00849	0.00720	0.39781	0.29570	
mannosidase *Rvi6*-avirulent	784	0.481	23	2.93	22	19	0.72655	0.01067	0.00629	1.98139	0.07949	
glucosidase *Rvi6*-virulent	533	0.571	24	4.5	35	18	0.767	0.0181	0.01152	1.93341	−0.27958	
glucosidase *Rvi6*-avirulent	533	0.568	64	12	55	48	0.9240	0.02697	0.02876	−0.21363	−0.45163	

*—*p* < 0.05.

**Table 3:** Neutral and non-neutral polymorphisms within *Rvi6*-virulent and -avirulent populations of *V. inaequalis* according to McDonald–Kreitman test.

Gene Region.	Neutral	Non-Neutral	Total
EF-1α	12	22	34
β-tubulin	17	17	34
mannosidase	26	3	29
glucosidase	23	48	71

**Table 4:** Estimators of gene flow and DNA divergence between *Rvi6*-virulent and -avirulent populations of *V. inaequalis*, obtained for single loci (EF-1α, β-tubulin, mannosidase and glucan-1,3-β-glucosidase) and concatenated data and minimal number of recombination events (*R_M_*) observed across the data.

	DNA Divergence between *Rvi6*-Virulent vs. *Rvi6*-Avirulent Populations			
	Gene Flow Estimates of Nei 1973	Average No. of Nucleotide Differentiations between Pops	Average Number of Nucleotide Substitutions per Site, between Pops Dxy	Minimum Number of Recombination Events [*Rm*]
EF-1α	Gst = 0.00025 Nm = 990.68	7.699	0.02291	20
β-tubulin	Gst = 0.13228 Nm = 1.64	3.912	0.01021	3
1,2-alpha-D-mannosidase	Gst = 0.01172 Nm = 21.08	7.792	0.00994	11
glucan-1,3-beta-glucosidase	Gst = 0.01324 Nm = 18.64	11.354	0.02239	12
concatened EF-1α and β-tubulin	Gst = 0.01068 Nm = 23.15	11.601	0.01614	23
concatenated mannosidase and glucosidase	Gst = 0.00344 Nm = 72.33	19.128	0.01482	23

**Table 5:** Estimates of molecular variance (AMOVA) and gene flow (F_ST_) obtained for two assumed populations of *V. inaequalis*: *Rvi6*-virulent and -avirulent and for the comparison between these two populations.

DNA Region and Groups of Populations	Among Groups	Among Populations within Groups	Among Individuals within Populations	F_ST_
EF-1α/*Rvi6*-virulent vs. *Rvi6*-avirulent	3.2	20.2	76.7	0.23
β-tubulin/*Rvi6*-virulent vs. *Rvi6*-avirulent	31.9	2.5	65.6	0.34
1,2-alpha-D-mannosidase/*Rvi6*-virulent vs. *Rvi6*-avirulent	2.6	13.5	83.9	0.16
glucan-1,3-beta-glucosidase/*Rvi6*-virulent vs. *Rvi6*-avirulent	0.6	7.7	91.7	0.08
EF-1α/*Rvi6*-virulent		23.0	77.0	0.23
EF-1α/*Rvi6*-avirulent		18.0	82.0	0.18
β-tubulin/*Rvi6*-virulent		10.2	89.8	0.10
β-tubulin/*Rvi6*-avirulent		22.0	78.1	0.22
1,2-alpha-D-mannosidase/*Rvi6*-virulent		19.6	80.4	0.20
1,2-alpha-D-mannosidase/*Rvi6*-avirulent		10.8	89.2	0.11
glucan-1,3-beta-glucosidase/*Rvi6*-virulent		17.8	82.2	0.18
glucan-1,3-beta-glucosidase/*Rvi6*-avirulent		3.4	96.6	0.03

**Table 6:** Estimation of index of association (rBARd) calculated between 100 strains of *V. inaequalis* and between two assumed groups of populations: *Rvi6*-virulent and -avirulent.

DNA Region and Compared Groups of Strains	Index of Association [*r*BAR*d*]
EF-1α all strains/two groups	0.15 *
β-tubulin all strains/two groups	0.3 *
1,2-alpha-D-mannosidase all strains/two groups	0.5 *
glucan-1,3-beta-glucosidase all strains/two groups	0.17 *
concatened EF-1α and β-tubulin, all strains/two groups	0.11
concatened mannosidase and glucosidase, all strains/two groups	0.13 *

*—*p* < 0.001.

**Table 7:** Primers used in the study.

Name of the Primer Set (Target Region)	Sequence of the Primer in 5′->3′ Orientation	Sequence of the Primer in 5′->3′ Orientation	Product Length in Basepairs	Reference
VNEFI-f/VNEFI-r (EF-1α)	ACTTGATCTACAAGTGCGGTG	AGGAGTCTCGAACTTCCAGAG	385	[14]
C/D (β-tubulin)	GAGGAATTCCCAGACCGTATGATG	GCTGGATCCTATTCTTTGGGTCGAACAT	436	[39]
cont189/675 (β-tubulin)	CACGGAAGATAGCGGAGCAAGTAA	GAGGAATTCCCAGACCGTATGATG	487	this study
VinManno1/ VinManno2	TTGCTCGCGTAACGGCTCCAGA	TTCGCTCATCGCAAATCCCCATAC	858	this study
VinGluco1/VinGluco2	TAAGCACGGCCATCACAACCTACG	CGCAGGCCCTCTAAATTCCAAACT	1006	this study

## Data Availability

All DNA sequences obtained during the study were deposited in the GenBank database at https://www.ncbi.nlm.nih.gov/genbank/.

## Supplementary Materials

The following are available online at https://www.mdpi.com/article/10.3390/pathogens10040447/s1, Supplementary File 1: The list of accession numbers assigned to sequences of *V. inaequalis* analyzed in the study and deposited in GenBank database.
